# ﻿*Bomareapastazensis* (Alstroemeriaceae), an exceptionally small new species from the eastern Andean slopes of Ecuador

**DOI:** 10.3897/phytokeys.235.110525

**Published:** 2023-11-10

**Authors:** John L. Clark, Alisson Fierro-Minda, Nolan Exe, Mia Johnson, Carrie M. Tribble, Lou Jost

**Affiliations:** 1 Marie Selby Botanical Gardens, 1534 Mound Street, Sarasota, FL 34236, USA Marie Selby Botanical Gardens Sarasota United States of America; 2 Herbario QUSF, Colegio de Ciencias Biológicas y Ambientales, Universidad San Francisco de Quito, USFQ, Quito, Ecuador Universidad San Francisco de Quito Quito Ecuador; 3 Herbario QCA, Escuela de Ciencias Biológicas, Pontificia Universidad Católica de Ecuador, Quito, Ecuador Pontificia Universidad Católica de Ecuador Quito Ecuador; 4 Reserva: The Youth Land Trust, Washington DC, USA Reserva: The Youth Land Trust Washington United States of America; 5 Reservas de Fundación EcoMinga, Fundación EcoMinga, Baños, Tungurahua, Ecuador Fundación EcoMinga Baños Ecuador; 6 School of Life Sciences, University of Hawai’i at Mānoa, Honolulu, HI, USA University of Hawaiʻi at Mānoa Honolulu United States of America

**Keywords:** Alstroemeriaceae, Andes, *Bomarea*, Ecuador, endemism, taxonomy

## Abstract

Recent field research on the eastern slopes of the Andes resulted in the discovery of a new species of *Bomarea* from the Cerro Candelaria Reserve in the Tungurahua province of Ecuador. *Bomareapastazensis* is the second smallest species in the genus and differs from the smallest by the presence of glutinous trichomes on the ovary, glabrous sepals, and greenish-yellow petals with purple spots. Based on IUCN guidelines, a preliminary conservation status is assigned as Vulnerable (VU).

## Abstract (es)

Recientes investigaciones de campo en las estribaciones orientales de los Andes dieron como resultado el descubrimiento de una nueva especie de *Bomarea* en la Reserva Cerro Candelaria en la provincia de Tungurahua en Ecuador. *Bomareapastazensis* es la segunda especie más pequeña del género y se diferencia por la presencia de tricomas glutinosos en el ovario, sépalos glabros y pétalos verdes con amarillo y manchas moradas. Con base en los lineamientos de la IUCN, se le asigna un estado preliminar de conservación de Vulnerable (VU).

## ﻿Introduction

There are more than 100 species of *Bomarea* Mirb. in South America (Hofreiter 2006) and 39 species in Ecuador (Harling and Neuendorf 2003). The description of *Bomareapastazensis* brings the total diversity to 40 species in Ecuador. The most recent monograph of *Bomarea* was provided by Hofreiter and Tillich (2002) and included an updated classification system based on Baker (1888) that recognized the following four subgenera: *Baccata* Hofr., *Bomarea* Baker, *Sphaerine* (Herb.) Baker, and *Wichuraea* (M. Roemer) Baker. More recently, molecular phylogenetic studies have failed to support the monophyly of these subgenera (Alzate et al. 2008b; Tribble et al. 2022), suggesting that many of the morphological characters that previously defined infrageneric classification are homoplastic or have evolved convergently. Alzate et al. (2008b) showed that traditionally recognized subgeneric ranks proposed by Baker (1888) and Hofreiter and Tillich (2002) are polyphyletic. Tribble et al. (2022) described three main clades within *Bomarea*, but no updated classification system has yet been proposed. In the present study we recognize the lack of strongly supported subgeneric ranks (Alzate et al. 2008b; Tribble et al. 2022) and discuss the traditional subgenus that most resembles *Bomareapastazensis*.

**Figure 1.**
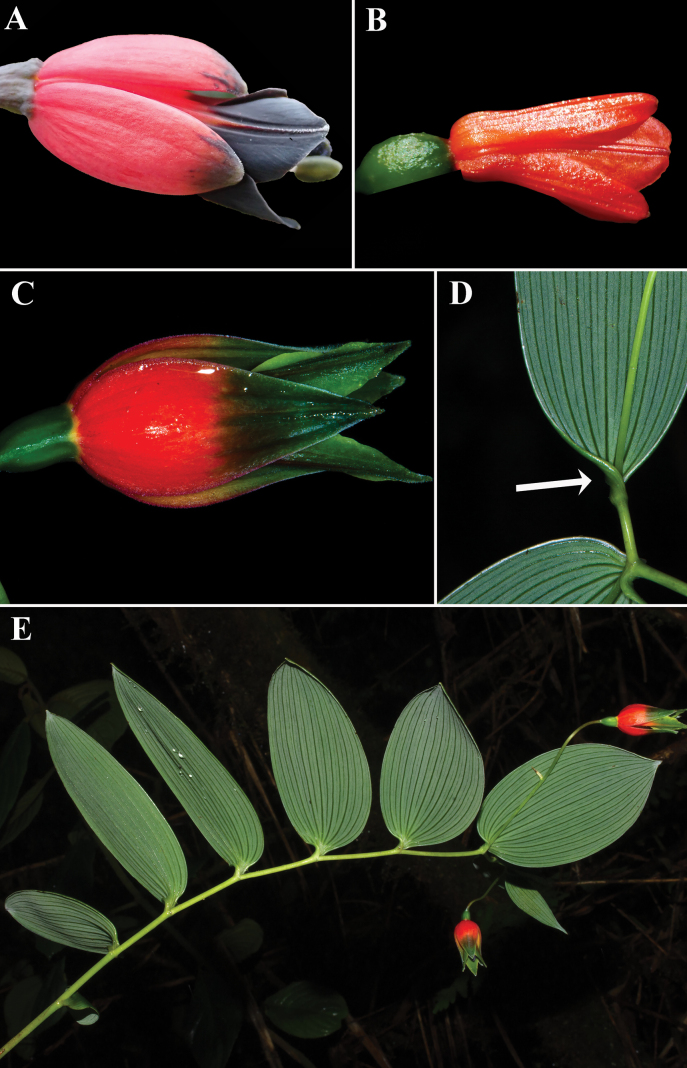
Some species of Ecuadorian BomareasubgenusSphaerine**A***Bomareabrachysepala***B***Bomareadistichifolia***C***Bomareahieronymi***D** resupinate leaf (rotated petiole indicated with white arrow) of *Bomareahieronymi***E** terrestrial habit (non-twining) of *Bomareahieronymi*. Photos **A, B** by N. Exe, **C–E** by J.L. Clark (*J.L. Clark 17350*).

**Figure 2.**
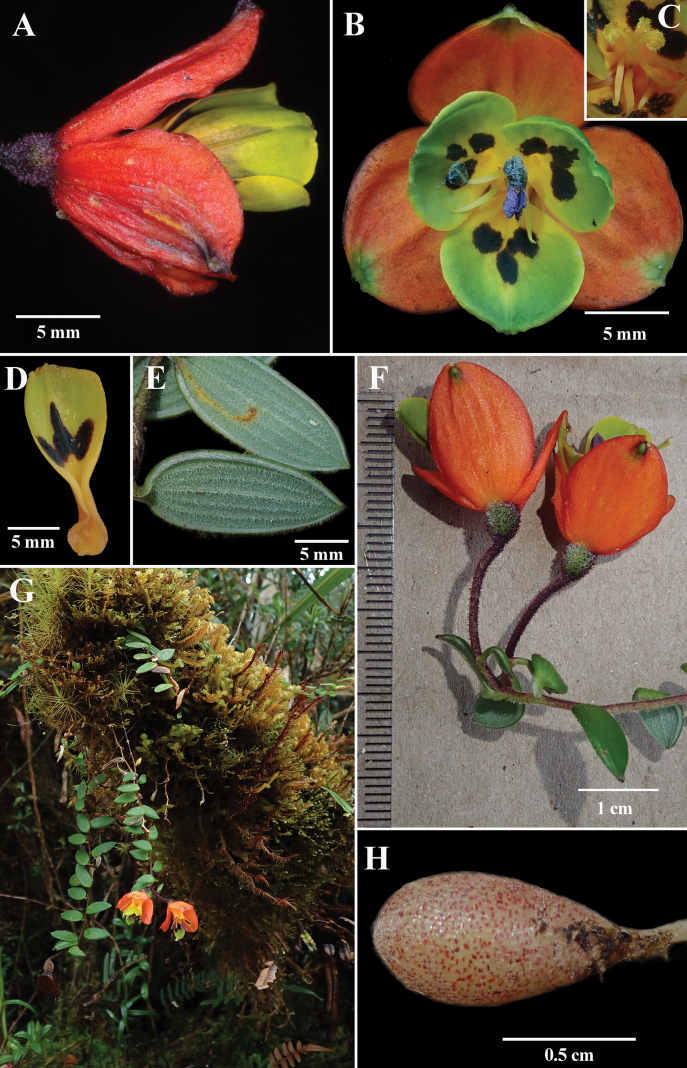
*Bomareapastazensis***A** lateral view of flower **B** front view of flower featuring mature androecium **C** mature gynoecium **D** petal **E** adaxial leaf surface **F** mature flowers with foliage **G** habitat **H** tuberous roots. Photos **A** by J.L. Clark (*J.L. Clark 14839*), **B–H** by N. Exe (*N. Exe et al. 2*).

*Bomareapastazensis* most closely resembles the morphologies associated with the subgenus Sphaerine because of the following characters (Hofreiter 2005): erect or hanging habit [mostly non-twining except for *B.coccinea* (Ruiz & Pav.) Baker] (Fig. 1E), resupinate leaves (Figs 1D, 2E), and inferior ovaries (Figs 1A–C, 2A). Recent molecular work suggests that *Sphaerine* is non-monophyletic and some morphological characteristics of this group are likely adaptations to environmental conditions (Alzate et al. 2008b; Tribble et al. 2022). The subgenus Sphaerine ranges from the northern Andes of Colombia and Venezuela to Bolivia. In Ecuador, the subgenus is distributed mainly on the eastern Andean slopes. A detailed taxonomic history and overview of *Sphaerine* was provided by Hofreiter (2006), including updated circumscriptions and two new species. The addition of *Bomareapastazensis* brings the total diversity of subgenus Sphaerine to 13 species.

Ecuador is the center of diversity for *Bomarea* (Hofreiter and Tillich 2002; Harling and Neuendorf 2003; Hofreiter 2005; Hofreiter and Rodríguez 2006; Alzate et al. 2008a), which is supported by the presence of 40 of the 120+ currently known species. The other species of BomareasubgenusSphaerine occurring in Ecuador (Hofreiter 2005; Hofreiter and Rodríguez 2006) are: *B.brachysepala* Benth. (Fig. 1A), *B.distichifolia* (Ruiz & Pav.) Baker (Fig. 1B), *B.hieronymi* Pax (Fig. 1), *B.linifolia* (Kunth) Baker, and *B.nervosa* (Herb.) Baker.

**Figure 3.**
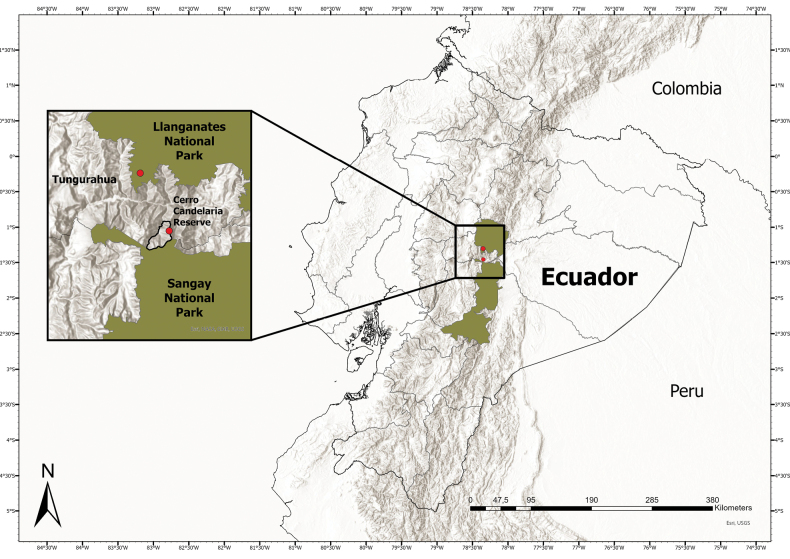
Distribution map of *Bomareapastazensis*. Red dots indicate collections or observations.

The Cerro Candelaria Reserve, owned by the Ecuadorian NGO Fundación EcoMinga, is within the upper Pastaza watershed, an area recently documented for high-levels of local endemism because of the presence of microclimates created by Amazon-Andean airflow currents and the irregular topography (Jost 2004). The Cerro Candelaria Reserve comprises 2800+ hectares, ranging in altitude from 1700 to 3860 m (Reyes-Puig et al. 2013). The private reserve is located within the Llanganates-Sangay corridor, which facilitates a protected corridor for biodiversity between the Llanganates and Sangay National Parks (Fig. 3).

## ﻿Materials and methods

Plants were photographed and collected during three separate field expeditions to the Cerro Candelaria Reserve by Clark in 2016 (Clark 2016), Exe and Johnson in January 2022, and Exe, Johnson, and Fierro-Minda in November 2022. Specimens were deposited at Pontificia Universidad Católica del Ecuador (QCA), Marie Selby Botanical Gardens (SEL), and Universidad de Guayaquil (GUAY). Digital images were taken of live specimens in the field using an Olympus TG5 and a Nikon D100 DSLR with a Nikon 105 mm lens and a Nikon SB-29s ring flash. Morphological observations and measurements were made from live collections, herbarium specimens, and digital images using the program ImageJ (Schneider et al. 2012).

We assessed the extinction risk of *Bomareapastazensis* following the IUCN Red List Categories and Criteria (IUCN 2012) and guidelines of the IUCN Standards and Petitions Committee (2022). We considered observations, collection localities, and population estimate from fieldwork. Species extent of occurrence (EOO) and area of occupancy (AOO) were calculated using GeoCAT (Bachman et al. 2011) with the default setting of 2 km^2^ grid.

## ﻿A note on terminology

Some botanical terminology has been used inconsistently in previous descriptions of *Bomarea*, and other terminology is specific to *Bomarea*. Here, we clarify our use of potentially confusing terms to ensure that our definitions are unambiguous while linking the description to other literature. Botanical glossaries usually refer to sepals as the outer whorl of the perianth and petals as the inner whorl of the perianth (e.g., Harris and Harris 2006; Pell and Angell 2016). In contrast, ‘tepal’ is used when describing cases where the outer and inner perianth whorls are identical or when perianth whorls are not readily differentiated. Some *Bomarea* taxonomists use the terms inner and outer tepals in their descriptions of the perianth (Hofreiter 2005; 2006; Hofreiter and Rodríguez 2006) and others use the terms sepals and petals (Harling and Neuendorf 2003). The description of *Bomareapastazensis* uses sepals and petals to provide greater specificity because the two perianth layers are easily differentiated. Furthermore, we use the terms adaxial and abaxial following the definition of these terms to refer to the portion of the leaf facing towards or away from, respectively, the structure-bearing organ (the stem) during development (Eckel 2011). When referring to the surface of resupinate leaves (rotating 180 degrees) of *Bomareapastazensis*, abaxial is the upper surface of the leaf, and adaxial is the lower surface of the leaf, terminology that is consistent with other *Bomarea* descriptions (Hofreiter and Lyshede 2006). Following Hofreiter (2005), we use the term “claw” to refer to the horn-like thickened sepal apex in many *Bomarea* species.

## ﻿Taxonomic treatment

### ﻿ Bomarea pastazensis

Taxon classificationPlantaeLilialesAlstroemeriaceae

J.L.Clark, Fierro-Minda & N.Exe sp. nov.

79BFC9D3-526F-5AEA-B9A1-7253EDCDC6F3

urn:lsid:ipni.org:names:77330582-1

Figs 2 , 4

#### Diagnosis.

Similar to *Bomareapumila* Griseb. ex Baker, differing in plant height reaching 10–14 cm (vs. 5–8 cm high in *B.pumila*), pubescent stem (vs. glabrous stem in *B.pumila*), the presence of ciliate leaf margin (vs. glabrous leaf margin in *B.pumila*), and the presence of dark spots on the petals (vs. no spotting on the petals in *B.pumila*).

#### Type.

**Ecuador. Tungurahua**: Cantón Baños, Parroquia Río Verde, Cerro Candelaria Reserve (Fundación EcoMinga), upper Pastaza watershed, 1°28'39.33"S, 78°17'53.61"W, 3642 m, 10 Mar 2016, *J.L. Clark 14839* (holotype: QCA! [245371]; isotype: SEL! [079072]).

#### Description.

Terrestrial or epiphytic herb. ***Rhizome*** short with multiple underground fusiform to globose root tubers, pale and heavily stippled with dull reddish-purple spots, 5–9 × 3–4 mm, surface striate, yellowish-brown with red spots (Fig. 2H). ***Stem*** erect, 10–14 cm long, ca. 0.13 cm in diameter, slender, terete, base pubescent, apex puberulous, internodes 0.6 cm long. ***Leaves*** alternate; blade ovate, 1.4–1.8 × 0.4–0.7 cm, base rounded to obtuse, apex acute, abaxially glabrous, adaxially light green, suffused with whitish-translucent trichomes, ca., 0.2 mm long, clustered along veins; blades with 5–7 prominent parallel veins, raised below and slightly raised above; margin hyaline, slightly revolute with unicellular trichomes to 0.2 mm long; petioles resupinate and canaliculate, 0.1–0.4 cm long, basal leaves reduced to scales. ***Flowers*** produced from the stem apex, usually one (rarely two) apical flower(s) per stem.; pedicels 1–1.4 cm long, terete, with brownish red to dark purple trichomes, bisexual, actinomorphic, epigynous. ***Sepals*** 3, 1.0–1.3 × 1.0–1.2 cm, each sepal with 6 parallel veins, broadly ovate, apically rounded, bright red to orange-red with a 1 mm green to black claw, inner and outer surfaces glabrous. ***Petals*** 3, basally constricted and caniculate, distally obovate to broadly spathulate, 1.5–1.6 × 0.6–0.7 cm, greenish yellow, puberulous at base, inner surface with 3 large reddish-brown spots, outer surface uniformly yellow (occasionally yellow suffused with red from the inner spots). ***Androecium*** of 6 free stamens, 0.8–0.9 cm long, thickened near center of filaments; anthers pseudo-basifixed, 0.3 × 0.1 cm, fusiform; pollen grains lilac. ***Gynoecium*** comprised of three fused carpels, ovary 0.5 × 0.4 cm with surface covered with glutinous trichomes, style ca. 1 cm long, stigma with three circinate lobes. ***Fruits*** not observed.

#### Ecology.

Found growing on *Sphagnum* and mossy cushions at ground level and epiphytically (up to 2.5 m high) in high elevation cloud forest and paramo (observed from 3235 to 3700 m). Plants growing in partially shaded areas to full sunlight. Stems erect to hanging, with flowers often found on or slightly above ground level. Flowers protandrous, commonly with one flower per stem but occasionally two.

#### Phenology.

Observed in flower in January, March, July, and November. Approximately 20 individual plants with mature flowers were located during an expedition in November of 2022 and fewer individuals with mature flowers were observed in January of 2022. Fruits not documented.

#### Etymology.

The specific epiphyte, *pastazensis*, reflects the watershed of the type locality that includes Río Pastaza and adjacent tributaries.

#### Distribution and preliminary assessment of conservation status.

*Bomareapastazensis* is endemic to the upper Pastaza watershed, located in the eastern Andean slopes of Ecuador. The first documented population was inside Cerro Candelaria Reserve in Tungurahua province (Fig. 3). The Cerro Candelaria Reserve is a private reserve of 2800+ hectares managed by Fundación EcoMinga. It is bordered on the north by EcoMinga’s Naturetrek Reserve, and the south by Parque Nacional Sangay. Along with Fundación EcoMinga’s Machay and Naturetrek Reserves to the north, it forms a protected corridor between Parque Nacional Sangay and Parque Nacional Llanganates. Cerro Candelaria Reserve was founded by Fundación EcoMinga in 2007 and financed by the World Land Trust (UK); the majority of the reserve is undisturbed by anthropogenic activity. A diverse flora and fauna has been recorded here, including many endemic species and species of high conservation priority (Jost 2004; Reyes-Puig et al. 2013), such as *Blakeaattenboroughii* Penneys & L.Jost (Melastomataceae) which is endemic to the lower elevation forests of Bosque Protector Cerro Candelaria. Several recently described species in the Orchidaceae share a similar geographic distribution to *Bomareapastazensis* (Fig. 3), include *Teagueiabarbeliana* L.Jost & Shepard, *T.puroana* L.Jost & Shepard, *T.kostoglouana* L.Jost & Shepard, *T.lizziefinchiana* L.Jost & Shepard, and *T.anitana* L.Jost & Shepard (Jost and Shepard 2011; 2017). Additional surveys in Cerro Candelaria and in the surrounding upper Rio Pastaza watershed are likely to yield many more undescribed species, and potentially additional populations of *Bomareapastazensis*. The distribution of this species highlights the importance of the Llanganates-Sangay corridor for allowing species gene flow and connectivity among populations (Ríos Alvear and Reyes-Puig 2015). A second documented population of *Bomareapastazensis* was provided by digital images from EcoMinga’s park guard Eduardo Peña (July 2023) in the Llanganates National Park, north of Río Pastaza (Fig. 3). We calculated the AOO=12 km^2^ from Peña’s observation and the type collection (Fig. 3). Based on the available information and according to the IUCN Red List criteria (IUCN 2012; IUCN Standards and Petitions Committee 2022), *Bomareapastazensis* is preliminarily assessed as Vulnerable (VU) based on a limited area of occupancy (IUCN criterion D2 where AOO <20 km^2^) and dependence on conservation efforts for its continued survival. The only documented populations of *Bomareapastazensis* are located inside protected areas, at elevations that are used for agriculture in unprotected parts of the Pastaza province. Effective conservation of this and the other unique species of the Pastaza watershed will require constant vigilance.

#### Comments.

*Bomareapastazensis* differs from other congeners by a distinctive pattern of three dark spots on the petals (Fig. 2D), widely opened flowers, and small size. *Bomareapumila* and *B.pastazensis* share similar traits such as their small size in comparison with the rest of *Bomarea* species, the presence of pubescence throughout their above-surface organs, and the color patterns of the flowers (Figs 2, 5; Table 1). *B.pastazensis* is slightly bigger in size (10–14 cm tall) than *B.pumila* (5–9 cm tall). In contrast, *B.pastazensis* is sparsely pubescent at the base of the sepals, while the sepals of *B.pumila* are uniformly densely pubescent (Fig. 5). The petals of *B.pumila* are not spotted, which differentiates it from *B.pastazensis* which has a three-spotted pattern on the petals (Fig. 2B). The two species are geographically isolated with *B.pastazensis* endemic to the Pastaza watershed on the eastern Andean slopes in central Ecuador (Tungurahua province) and *B.pumila* from Central Peru to northern Bolivia. Table 1 provides a summary of distribution and useful characters for differentiating *B.pastazensis* and *B.pumila*.

**Table 1.** General geographic distribution (names in parentheses indicate Ecuadorian province) and comparison of morphological characters between *Bomareapastazensis* and *B.pumila*.

	*Bomareapumila*	*Bomareapastazensis*
Habit	erect	erect to hanging
Plant height	5–9 cm long	10–14 cm long
Stem surface	glabrous	pubescent
Leaf – relative size	leaves not uniform on stem; central leaves relatively larger compared with basal and apical leaves	leaves relatively uniform on stem
Leaf margin	glabrous	ciliate
Pedicel length	1.5–4 cm long	1–1.4 cm long
Flower length	0.8–1.5 cm long	1.5–1.6 cm long
Flower width during anthesis	2–3 cm wide	1.5–2 cm wide
Sepal surface	conspicuously pubescent	sparsely pubescent at base only
Sepal horn	white to reddish horn at apex	green to black horn at apex
Petal coloration (=inner tepals)	yellow with a red stripe and green tip.	green suffused with yellow with three prominent red spots at the base
Distribution	Peru and Bolivia	Ecuador (Tungurahua)

**Figure 4.**
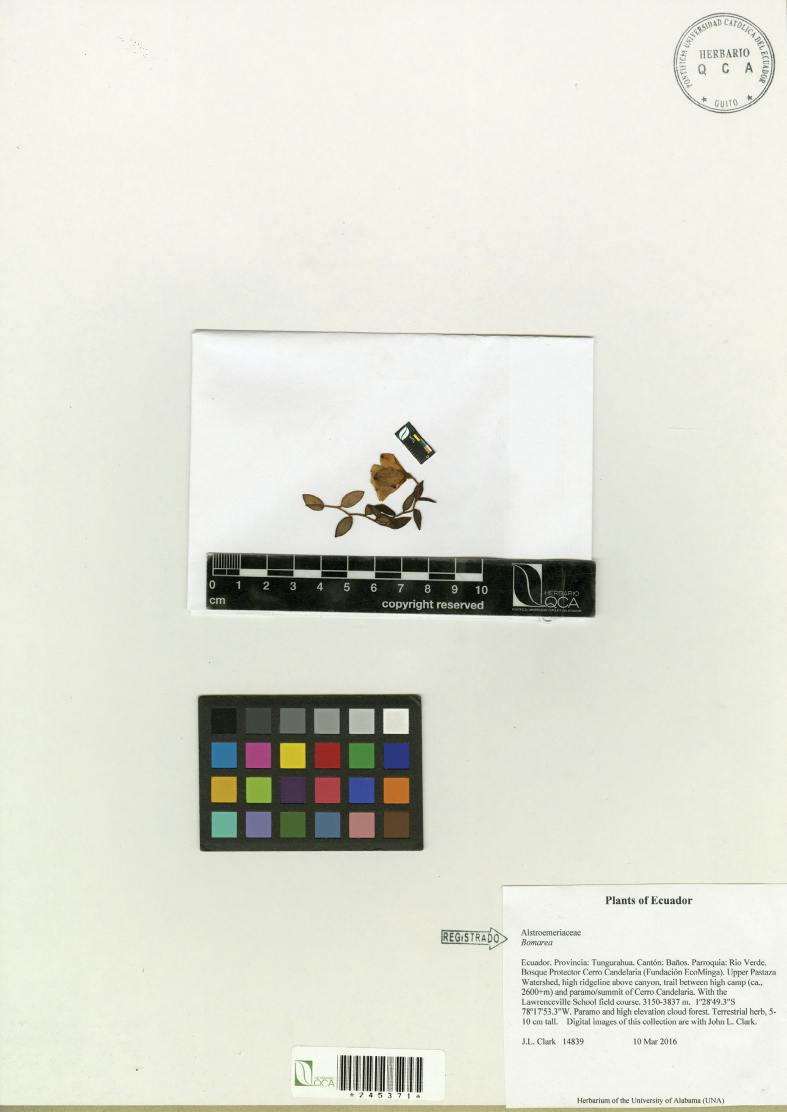
Holotype of *Bomareapastazensis*, *J.L. Clark 14839* (QCA).

**Figure 5.**
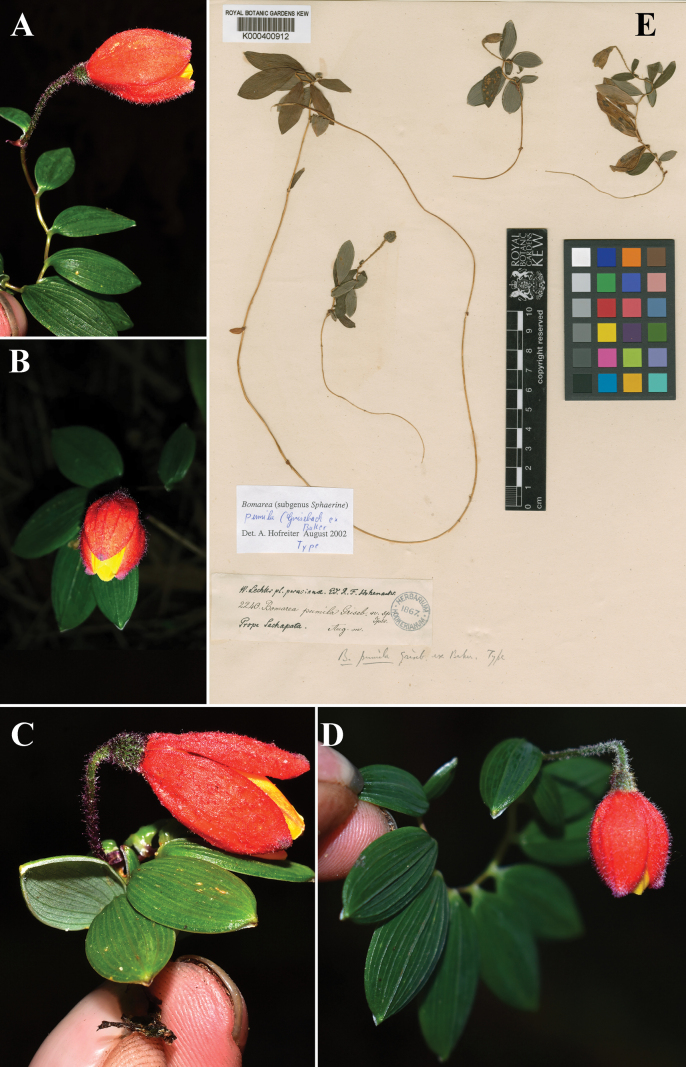
*Bomareapumila***A–D** field images from Peru **E** syntype (K! [K000400912]) of *Bomareapumila* of *W. Lechler 2240* from Sachapata, Peru. Photos **A–D** from Lucely L. Vilca Bustamente **E** from The Herbarium Catalogue, Royal Botanic Gardens, Kew. The Board of Trustees of the RBG, Kew.

#### Additional specimens examined.

**Ecuador. Tungurahua**: Cantón Baños, Parroquia Río Verde, Bosque Protector Cerro Candelaria (Fundación EcoMinga), upper Pastaza watershed, Cerro Candelaria summit trail, just below paramo, 1°28'39.33"S, 78°17'53.61"W, 3150–3827 m, 1 Jan 2022, *N. Exe, M. Johnson & A. Fierro-Minda 2* (GUAY).

## Supplementary Material

XML Treatment for Bomarea pastazensis

## ﻿Additional information

### Author ORCIDs

John L. Clark https://orcid.org/0000-0002-1414-6380

Alisson Fierro-Minda https://orcid.org/0000-0001-7169-2989

Nolan Exe https://orcid.org/0000-0001-8847-7978

Mia Johnson https://orcid.org/0000-0002-4822-0606

Carrie M. Tribble https://orcid.org/0000-0001-7263-7885

Lou Jost https://orcid.org/0000-0002-5850-0716

### Data availability

All of the data that support the findings of this study are available in the main text.
